# Response of blacktip reef sharks *Carcharhinus melanopterus* to shark bite mitigation products

**DOI:** 10.1038/s41598-020-60062-x

**Published:** 2020-02-27

**Authors:** Madeline Thiele, Johann Mourier, Yannis Papastamatiou, Laurent Ballesta, Eric Chateauminois, Charlie Huveneers

**Affiliations:** 1 Southern Shark Ecology Group, College of Science and Engineering, Flinders University, Bedford Park, SA 5042 Australia; 2 MARBEC, Univ Montpellier, CNRS, IFREMER, IRD, Sète, France; 3 EPHE, PSL Research University, CRIOBE USR3278 EPHECNRS-UPVD, 66860 Perpignan, France; 4 Labex Corail, CRIOBE, 98729 Moorea, French Polynesia; 5 Department of Biological Sciences, Florida International University, North Miami, Florida USA; 6 Andromede Oceanologie, Place Cassan, 34280 Carnon, France; 7 Centre Securite Requin, 25 F avenue des artisans, zone Artisanale de la Pointe des Chateaux, 97436 Saint-Leu, Reunion Island France

**Keywords:** Behavioural ecology, Animal behaviour

## Abstract

Globally, the frequency of shark bites is rising, resulting in an increasing demand for shark deterrents and measures to lessen the impact of shark bites on humans. Most existing shark protection measures are designed to reduce the probability of a bite, but fabrics that minimise injuries when a shark bite occurs can also be used as mitigation devices. Here, we assessed the ability of the Ocean Guardian Scuba7 and Kevlar material to reduce the likelihood of blacktip reef sharks, *Carcharhinus melanopterus*, from feeding, and to minimise injuries from shark bites. Sharks were enticed to consume a small piece of local reef fish (bait) placed between the two Scuba7 electrodes with the deterrents randomly being turned on or kept off. In the second experiment, the bait was attached to a small pouch made of either standard neoprene or neoprene with a protective layer of Kevlar around it. The Scuba7 reduced the proportion of baits being taken by 67%, (from 100% during control trials to 33%). Sharks also took more time to take the bait when the device was active (165 ± 20.40 s vs. 38.9 ± 3.35 s), approached at a greater distance (80.98 ± 1.72 cm vs. 38.88 ± 3.20 cm) and made a greater number of approaches per trial (19.38 ± 2.29 vs. 3.62 ± 0.53) than when the Scuba7 was inactive. The sizes of punctures from shark bites were significantly smaller on neoprene with Kevlar compared to standard neoprene (3.64 ± 0.26 mm vs. 5.88 ± 0.29 mm). The number of punctures was also fewer when Kevlar was used (14.92 ± 3.16 vs. 74.1 ± 12.44). Overall, the Ocean Guardian Scuba7 and Kevlar reduced the impact of blacktip reef shark bites. These findings may help consumers make informed decisions when purchasing shark deterring and protective products.

## Introduction

Although shark-human interactions remain rare and unlikely events, their frequency has been increasing globally since the 1980s^1,2^. The primary cause of this increase is unknown, but factors typically listed include growth in human population and increased ocean use^3^, habitat destruction and modification, degrading water quality, changing weather patterns and climate change, a change in the abundance and distribution of prey (potentially shifting shark distributions into more human populated areas), and increasing shark abundance^1,4,5^. Whilst increasing, the irregular occurrence of shark bites hinders our ability to evaluate the relative importance of any of these potential causes (but see Afonso, *et al*.^4^ and Meyer, *et al*.^6^). However, as the number of people undertaking aquatic activities continues to rise and shark conservation actions improve, it is likely that shark bite incidents will increase^7^. Whilst irregular, the gruesome nature and potential lethality of shark bites is a focal point for traditional and social media, and entertainment (e.g., films and cartoons)^8,9^. This exaggerates the public’s perceived risk of shark bites^10^, leading to public pressure for the development and implementation of shark bite mitigation measures.

Shark bite mitigation measures are generally categorised in two broad approaches: area protection and personal protection^11,12^. The former aims to decrease the spatio-temporal overlap between humans and sharks, i.e. how frequently humans and sharks are in close proximity (e.g., the shark spotters program^13^). However, limitations of area protection strategies, such as weather conditions impacting effectiveness^14^ or inapplicability in some regions, led to a range of personal shark deterrent devices being developed to deter sharks from approaching humans^12^. The increasing demand for personal shark deterrents has led to the development of several new products, but few have been scientifically tested. Out of those tested, fewer have shown to significantly affect shark behaviour and reduce probability of shark bites (but see^12,15,16^). Most studies have also focused on white sharks, *Carcharodon carcharias*, because it is the species responsible for the most fatal shark bites^5^. However, at least 12 other species are responsible for shark bites^5^, and while bites from most of these other species are not fatal, they can still cause severe injuries and have negative implications to public perception of sharks. Mitigation of shark-human conflict and testing of shark deterrents should therefore not only focus on those species responsible for fatal bites, but should expand to other species.

When shark bites cannot be prevented by area protection or personal deterrents, the ability to reduce the severity of injuries can help in alleviating negative shark-human interactions. One of the most notable pieces of equipment to reduce wounds from shark bites is the chainmail suit developed in the 1980s. The suit seemingly reduced wounds from shark bites and is worn by divers undertaking shark feeding during wildlife tourism^17^. However, its heavy weight and lack of flexibility make it impractical in many situations (e.g., surfing, snorkelling) and only usable in limited diving situations. Recent material development has enabled the incorporation of puncture-resistant fabrics with neoprene, e.g., Kevlar. While these fabrics have been shown to reduce cuts^18,19^, their ability to withstand shark bites and reduce injuries is unknown.

This study aimed to test a personal shark deterrent (Ocean Guardian Scuba7; hereafter referred to as Scuba7) and to test a new material proposed to reduced injuries from shark bites (Kevlar). The Scuba7 is one of several products from Ocean Guardian aimed at deterring sharks by overwhelming their ampullae of Lorenzini^12^. The Scuba7 differs from other products in the size and configuration of electrodes which are placed on the ankle and tank of the diver (*vs* the Freedom7 where one of the electrode streams ~1.5 m behind the diver or snorkeler). Previous studies assessing the ability of electric deterrents to reduce the probability of shark bites have focused on white sharks, with testing predominantly occurring when the number of sharks present was relatively small, e.g., 1–4^12,15,16^. The response to an electric deterrent can, however, vary between species. In addition, competition can change behaviour and hierarchy in sharks^20^ and decrease the efficacy of magnets as shark deterrents^21^. Testing the effectiveness of electric deterrents on a different species and with a large number of sharks present, potentially affecting shark motivation through competition, will provide a better understanding of electric deterrent efficacy. Kevlar is a material derived from the aromatic polyamide family, which makes it strong, lightweight, flexible, and highly resistant to punctures and lacerations. Previous studies have shown the effectiveness and strength of Kevlar in hypervelocity and high impact situations, resulting in this material being used in bumper shields and bulletproof clothing^22,23^. These properties have the potential to provide better protection for ocean-users when a layer of Kevlar is added onto a standard neoprene wetsuit. Here, we tested the ability of the Scuba7 to prevent a medium-bodied shark species from feeding on a piece of fish and whether the addition of Kevlar on neoprene can reduce injuries such as those occurring when fishers or scientists handle sharks, or during scuba-diving and wildlife tourism activities.

## Methods

### Study location and species

The blacktip reef shark is a medium-sized shark, up to 1.5 m total length, and is widely distributed on Indo-West Pacific coral reefs^24^. The species mostly inhabits shallow reefs and sand-flats of atolls and high islands^25–28^ and occasionally non-reef environments^29^. Blacktip reef sharks are the subject of wildlife tourism, for example in Mo’orea, French Polynesia^30^, and they are often encountered by scuba-divers and snorkelers and during wildlife tourism operations targeting other species (e.g., bull sharks in Fiji^31^; lemon sharks in French Polynesia^32^; fish feeding in Palau; M. Thiele personal observation). These operations often use provisioning to attract sharks in close vicinity of tourists, which has resulted in accidental bites^33,34^.

Testing was undertaken on the top of the reef crest at the South pass of Fakarava Atoll, in the Tuamotu archipelago of French Polynesia (−16°52′S, −145°46′W), holding a population of ~100 blacktip reef sharks^35^. At this site, blacktip reef sharks are fed pieces of meat and fish daily when food is prepared for guests of the Tetamanu village, resulting in up to 30 blacktip reef sharks simultaneously frequenting and being fed next to the food preparation area.

### Field testing

We conducted tests on two products to assess the ability of mitigating bites from blacktip reef sharks. First, we tested the ability of an electric field-based deterrent developed for divers to repel sharks (i.e., the Scuba7 manufactured by Ocean Guardian). Second, we tested the ability of a layer of Kevlar glued to neoprene wetsuit material (hereafter referred to as Kevlar-neoprene) to reduce punctures or tears.

#### Scuba7 testing

**Figure 1:**
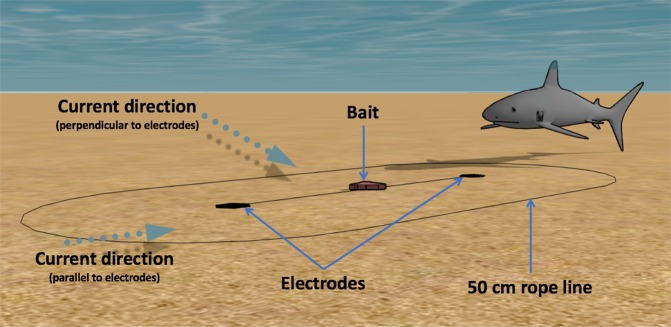
Diagram showing the experimental set-up and the two dominant current directions during the trials. Image drawn by the authors using SketchUp (www.sketchup.com).

The Scuba7 was deployed on the seabed (80% sand and 20% rocks and broken corals) in ~75 cm of water between 0900 and 1200 hours during seven consecutive days in June 2017. A coloured rope was placed 50 cm around the Scuba7 to facilitate estimates of the distance between sharks and the electrodes (Fig. 1).

Each day, we conducted 12 trials; six with the Scuba7 active and six with the Scuba7 inactive (control). Trials were block randomised so that the status of the Scuba7 (on or off) was randomly selected before each set of two trials. Status of the Scuba7 was confirmed by a green LED light present when turned on. This indicator was not covered during the trials so that the status of the device could be confirmed during the trials. The LED light was not expected to affect sharks as trials occurred during daylight reducing the relative intensity of the LED light, i.e. the intensity of the green light was not strong compared to normal sunlight. During each trial a small piece of local reef fish (bait) was placed between the two electrodes (85 cm from each electrode) using a long pole. Experimenters were not in the water during the trials and stood on a platform ~3 m from the experimental equipment and therefore did not influence shark behaviour. Each trial lasted three minutes or until a shark took the bait. Trial duration was short because of the large number of blacktip reef sharks present and their conditioning to being fed at that location resulting in sharks quickly taking bait. Preliminary tests showed that a bait lasts less than one minute before being consumed by a shark when the Scuba7 is absent. Trials were filmed by a GoPro Hero3+ video camera and footage was viewed using a VLC player. Coding of the video footage was analysed in a single-blind fashion because the coder did not participate in the trials and had no prior knowledge of the status of the Scuba7 when coding videos. Individual sharks could not be identified due to the lack of distinguishable markings or scars and because most sharks were of similar length. Instead, we recorded the maximum number of sharks during each trial within 30 m of the Scuba7. We used the following terminology to describe shark behaviour and code the videos, modified from previous studies^12,15^:

Approach: a directed swim towards the experimental set-up (each time a shark veered away from the bait and swam back, we classified it as a new approach);

Approach direction: whether sharks approach the Scuba7 from parallel to the dipole axis or perpendicular to the dipole axis;

Distance to electrode: distance from the sharks’ snout, where the shark’s sensory organs targeted by the deterrents (ampullae of Lorenzini) are located, to the closest electrode. Distance was visually estimated to the nearest 10 cm using a rope placed at a known length (50 cm) around the Scuba7 (Fig. 1); and

Reaction: a behavioural reaction from an individual shark towards the experimental set-up (e.g., tail flick, muscle spasm, head shake, fast direction change).

#### Kevlar-neoprene testing

We tested the Kevlar-neoprene between 0900 and 1200 hours on four days in June 2018. The Kevlar-neoprene consisted of ~1 mm Kevlar knitted sheets (19.5% Kevlar, 70% nylon, 10.5% spandex) glued on both sides of standard neoprene. Sections of Kevlar-neoprene and standard neoprene (without Kevlar) were sawed into 15 × 30 cm pouches on which pieces of meat or local reef fish were attached to entice blacktip reef sharks to bite the neoprene. We also had two neoprene thicknesses for each neoprene type: 5 mm and 3 mm. Neoprene pouches were left in the water until a shark bit the neoprene. Although bite intensity was not recorded, we ensured homogeneity of bites across the material tested by leaving the neoprene until a bite with similar intensity occurred, i.e. head shakes with sharks dragging the pouch in the water, as determined by the senior author (CH). Damages on the neoprene was quantified by a coder who was not present during the trials.

### Statistical analysis

#### Scuba7 testing

We investigated the effect of the Scuba7 on the following response variables: whether the bait was taken, time taken to take the bait, distance to electrode, number of approaches, and whether a reaction was observed. Following the method in Huveneers *et al*.^15^, we minimised potential temporal correlation by testing the effects of the Scuba7 on all response variables using a generalised linear mixed-effects model (GLMM) with *Day* coded as a random effect, the natural logarithm of the maximum number of sharks as offset, and the status of the Scuba7 (on or off) and the approach direction as fixed effects. Current direction was initially included as a fixed factor but was later removed from the model due to strong correlation with approach direction. Dominant current direction was perpendicular to the dipole axis on 4 of the 7 trial days and parallel to the dipole axis on the remaining days, ensuring a relatively balanced current direction across the study period. The error structure of GLMM corrects for non-independence of statistical units due to shared temporal structure and permits the random effects variance explained at different levels of clustering to be decomposed. The inclusion of *Day* as a random effect enabled the analysis to account for the lack of independence in behaviour within each Day. We determined the most appropriate statistical family and error distribution for each analysis by examining the distribution of the response variable and visually inspecting the residuals for the saturated models. Models were fitted with a gaussian distribution for all parameters tested, except to test the effect of the Scuba7 on the likelihood of the bait being taken for which a binomial distribution with logit link function was used. The number of approaches was scaled and centred prior to fitting the GLMM. The full model was dredged, creating a set of new models containing all possible combinations of the factors. These were ranked on decreasing model fit, using Akaike’s information criterion corrected for small sample size (AIC_c_)^36^. The bias-corrected relative weight of evidence for each model, given the data and the suite of candidate models considered, was the AIC_*c*_ weight; the smaller the weight, the lower its contribution to parameter estimates^36^.

#### Kevlar-neoprene testing

We also used a GLMM to test whether the following response variables were affected by the Kevlar-neoprene: number of punctures (regardless of whether they went through the neoprene or not); whether a puncture went through the neoprene (i.e., hole); and the length of the hole. *Day* was again coded as a random effect, with the neoprene type and thickness included as fixed effects.

All models were undertaken in R (v.3.5.0) using the lme and glmer function in the *nlme* and *lme4* packages^37,38^.

### Electric field modelling

The effects of undertaking the trials in shallow water was investigated by modelling the electric field propagation across different situations, e.g. in shallow or open water. The electric field was modelled as a dielectric dipole, where each conductive electrode was considered a point load with a charge of q and −q at 1.7 m from each other. Each charge delivers a potential scalar field *V*_*q*_ (Eq. 1):

1 $${V}_{q}({\rm{d}})=\frac{q}{4\pi {\varepsilon }_{0}{\varepsilon }_{r}}\frac{1}{d}$$

where ${\varepsilon }_{0}$ is the permittivity of the empty space; ${\varepsilon }_{r}$ the relative permittivity of the considered material; and d the distance from the charge q.

The total electric field delivered by the dipole [q, −q] is then calculated by adding the potential scalar field of each electrode, and applying the classic electromagnetism results derived from the Poisson equation (Eqs. 2 and 3):

2 $$\overrightarrow{E}({\rm{r}})=-\,{\rm{grad}}\,({V}_{tot})$$

3 $$\overrightarrow{E}={E}_{r}\overrightarrow{{U}_{r}}+{E}_{\theta }\overrightarrow{{U}_{\theta }}$$

$${E}_{r}=K[\frac{r-a\,\cos \,\theta }{{({a}^{2}+{r}^{2}-2ar\cos \theta )}^{3/2}}-\frac{r+a\,\cos \,\theta }{{({a}^{2}+{r}^{2}+2ar\cos \theta )}^{3/2}}]$$

$${E}_{\theta }=K[\frac{a\,\sin \,\theta }{{({a}^{2}+{r}^{2}-2ar\cos \theta )}^{3/2}}+\frac{a\,\sin \,\theta }{{({a}^{2}+{r}^{2}+2ar\cos \theta )}^{3/2}}]$$

with $$K=\frac{q}{4\pi {\varepsilon }_{0}{\varepsilon }_{r}}$$

where ${V}_{tot}$ is the total scalar field; r the distance from mid-point between the electrode; $\overrightarrow{{U}_{r}}$ and $\overrightarrow{{U}_{\theta }}$ the polar projection axes; and $a$ the distance between the electrode and mid-point between the electrodes. This provides the electric field delivered by the Scuba7 in open water.

This electric field was then modified to account for the possible effects of the surface and substrate using the method of image charges. This method states that, for a given distribution of charges, the solution of the Poisson equation is unique. Using this principle, we can add some fictive charges to simulate the presence of a non-conductive field (air or seabed) and compare their effect on the electric field. However, this method does not allow us to model the presence of the surface and seabed concurrently. The electric field propagation was therefore modelled for the following situations:

1. Mid-water: Scuba7 away from the surface or the seabed;
2. Surface: Scuba7 was 70 cm from the surface which is considered to be non-conductive. The effect of the seabed was ignored;
3. Bottom non-conductive: Scuba7 was 5 cm from the seabed which is considered to be non-conductive. The effect of the surface was ignored; and
4. Bottom conductive: Scuba7 was 5 cm from the seabed which is considered to be conductive. The effect of the surface was ignored.

Cases (3) and (4) give us information about the effect of the conductive parameter of the seabed.

The selected distances to the surface and seabed replicated the position of the Scuba7 in the trials undertaken whereby water depth was ~75 cm with the electrodes being ~5 cm above the seabed. For each situation, the electric field propagation is represented along the horizontal plane positioned 20 cm above the seabed (Z = −55 cm, which is the average swimming depth of sharks during the trials) and in the vertical plane going through the electrodes.

Using these models, we calculated the field strength that sharks were exposed to when crossing through the dipole axis along three trajectories going through the electrode mid-point: parallel, perpendicular, and diagonally. This allowed us to compare the field strengths that sharks were exposed to accounting for the effects of the surface and substrate as well as the effect of approach direction.

## Results

### Scuba7 testing

We conducted a total of 84 trials (42 with the Scuba7 active and 42 with the Scuba7 inactive) during which up to 24 blacktip reef sharks were sighted within 30 m of the bait. We recorded 966 approaches, with the mean (±standard error) distance between sharks and an electrode being 74.36 ± 1.61 cm (range: 0–220 cm).

**Figure 2:**
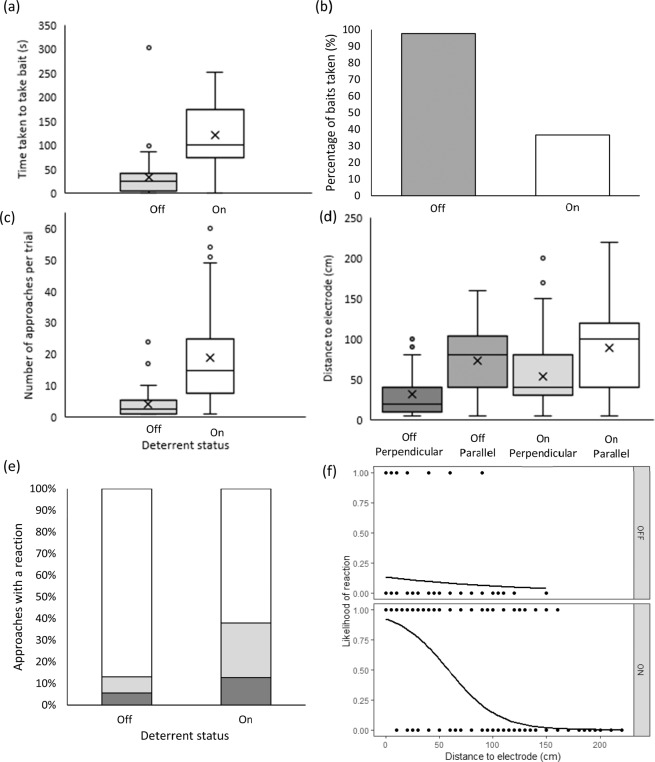
Effects of the Scuba7 on blacktip reef sharks (*Carcharhinus melanopterus*) on (**a**) time taken to take the bait; (**b**) percentage of baits taken; (**c**) number of approaches per trial; (**d**) distance to the electrode; (**e**) body reactions (white = approaches with no reaction, light grey = reaction during outside approaches, darker grey = reaction during in-between approaches); and (**f**) logistic regression of the likelihood of a reaction occurring as distance from electrode increases for trials when the Scuba7 was off (top) and on (bottom). Mean values are indicated by the crosses; median by the horizontal bar; the length of the box is the inter-quartile range; whiskers represent quartiles; and circles are extreme values.

The status of the Scuba7 significantly affected all response variables investigated: whether the bait was taken, time taken to take the bait, distance to electrode, number of approaches, and whether a reaction was observed (Table 1; Fig. 2a–f; Tables S1–5). When the Scuba7 was inactive, sharks took the bait during all trials in an average of 38.9 ± 3.35 s. In contrast, sharks took the bait on 33% of the trials and took about four times longer (165 ± 20.40 s) when the Scuba7 was active (Fig. 2a,b). This resulted in a greater number of approaches per trial (3.62 ± 0.53 vs. 19.38 ± 2.29 when the Scuba7 was turned off or on respectively), which also increased with the number of sharks present during the trials (Fig. 2c; Table S3). The Scuba7 also affected the minimum distance between sharks and electrode, which doubled from 38.88 ± 3.20 cm to 80.98 ± 1.72 cm. The minimum distance was also affected by approach direction, with sharks getting closer when approaching perpendicular to the dipole axis in-between the electrodes (40.28 ± 2.62 cm) compared to in line with the dipole axis (84.95 ± 1.78 cm) (Fig. 2d). Each time a shark consumed the bait with the Scuba7 active, approach direction was perpendicular to the dipole axis and between the electrodes.

**Table 1:** Estimated deterrent level coefficients (*β*) and their standard errors (SE), *z*-values of factors included in the top-ranked model (indicated for each variable), and the individual coefficient Type I error estimate (*P*) when relevant.

Level	*β*	SE	*z*	*P*
*Distance*				
intercept	3.46	0.51	6.76	
Deterrent	3.55	0.40	8.88	
Approach direction	3.14	0.46	6.79	
Deterrent* Approach direction	−1.09	0.53	−2.05	
*Time*				
Intercept	−0.82	0.09	−9.28	
Deterrent	1.64	0.12	13.12	
*Number of approaches*				
Intercept	−1.18	0.19	−6.25	
Deterrent	1.77	0.17	10.23	
*Bait consumption*				
Intercept	20.40	496.71	0.04	
Deterrent	−23.91	496.71	−0.05	
*Reaction*				
Intercept	−1.53	0.46	−3.35	0.001
Deterrent	6.03	0.67	9.05	<0.001
Approach distance	−0.07	0.01	−5.94	<0.001
Approach direction	0.71	0.69	1.03	0.304
Deterrent* Approach direction	−1.99	0.78	−2.55	0.011

All models include *Day* as a random effect. Baselines are Scuba7 off and approaches in-between electrodes.

We observed reactions 322 times at an average distance of 40.68 ± 1.84 cm (range: 0–160 cm) from an electrode, with most reactions (95%) occurring when the Scuba7 was active. The likelihood of reaction was affected by the combination of the deterrent status and approach direction (Table S5). When the Scuba7 was active, a reaction was more likely to occur when approaching in line with the dipole axis compared to perpendicular (66% vs. 34% respectively), but this effect was no longer apparent when the Scuba7 was inactive (47% vs 53%) (Fig. 2e). The likelihood of a reaction increased as sharks got closer to the electrode, but this was mostly driven by trials when the Scuba7 was active (Table S5; Fig. 2f).

### Kevlar-neoprene testing

**Figure 3:**
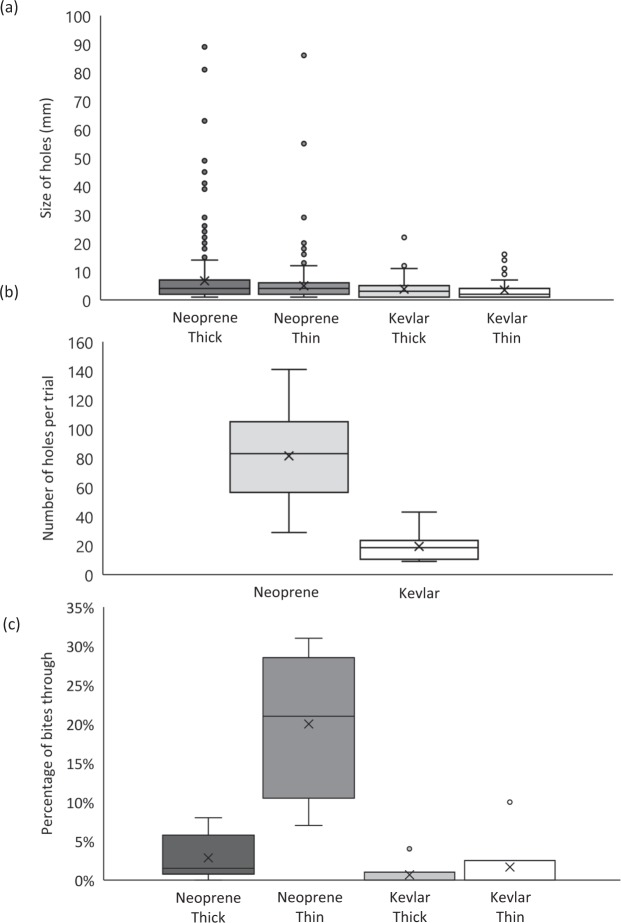
Damage of blacktip reef shark (*Carcharhinus melanopterus*) bites on standard and Kevlar-neoprene, based on (**a**) size of the holes; (**b**) number of holes per trial; and (**c**) percentage of holes that went through the fabric.

We obtained 22 bites, 12 on Kevlar-neoprene (six on 5 mm, six on 3 mm) and 10 bites on standard neoprene (six on 5 mm, four on 3 mm). The size of holes was significantly smaller when Kevlar-neoprene was used (3.64 ± 0.26 mm) compared to standard neoprene (5.88 ± 0.29 mm; t-value: 3.68; P < 0.0001), but was not affected by neoprene thickness (t-value: −0.29; P = 0.77) or the interaction between neoprene type and thickness (t-value: −1.12; P = 0.26) (Table 2; Fig. 3a), even though both these were included in the top-ranked model (Table S6). In the case of the number of holes, neoprene type was the only factor included in the top-ranked model (*w*AIC_c_ = 0.76; Table S7). The number of holes was significantly less when Kevlar-neoprene was used (14.92 ± 3.16) compared to standard neoprene (74.1 ± 12.44) (Table 2; Fig. 3b). The proportion of holes that went through the fabric was the only variable affected by neoprene thickness, with the interaction between neoprene type and thickness being included in the top-ranked model and having the largest coefficient (Table 2; *w*AIC_c_ = 0.99; Table S8). The proportion of holes through the thick fabric was similar between the standard and Kevlar-neoprene (2.8 ± 1.2% and 0.7 ± 0.7% respectively), but differed in the thin fabric, where the standard neoprene (20.0 ± 4.9%) had more holes through the fabric than the Kevlar-neoprene (1.7 ± 1.7%) (Fig. 3c).

**Table 2:** Estimated deterrent level coefficients (*β*) and their standard errors (SE), *z*-values of factors included in the top-ranked model (indicated for each variable), and the individual coefficient Type I error estimate (*P*). Baselines are Kevlar-neoprene and thick neoprene.

Level	*β*	SE	*z*	*P*
*Size of holes*				
intercept	3.77	0.70	5.39	<0.001
Fabric type	2.90	0.79	3.68	<0.001
Fabric thickness	−0.33	1.12	−0.29	0.770
Fabric*Thickness	−1.40	1.24	−1.12	0.261
*Number of holes*				
intercept	14.92	7.97	1.87	0.076
Fabric type	59.18	11.83	5.01	<0.001
*Proportion of holes punctured*				
intercept	0.01	0.02	0.33	0.744
Fabric type	0.02	0.03	0.76	0.455
Fabric thickness	0.01	0.03	0.35	0.729
Fabric*Thickness	0.16	0.04	3.80	0.001

### Electric field modelling

**Figure 4:**
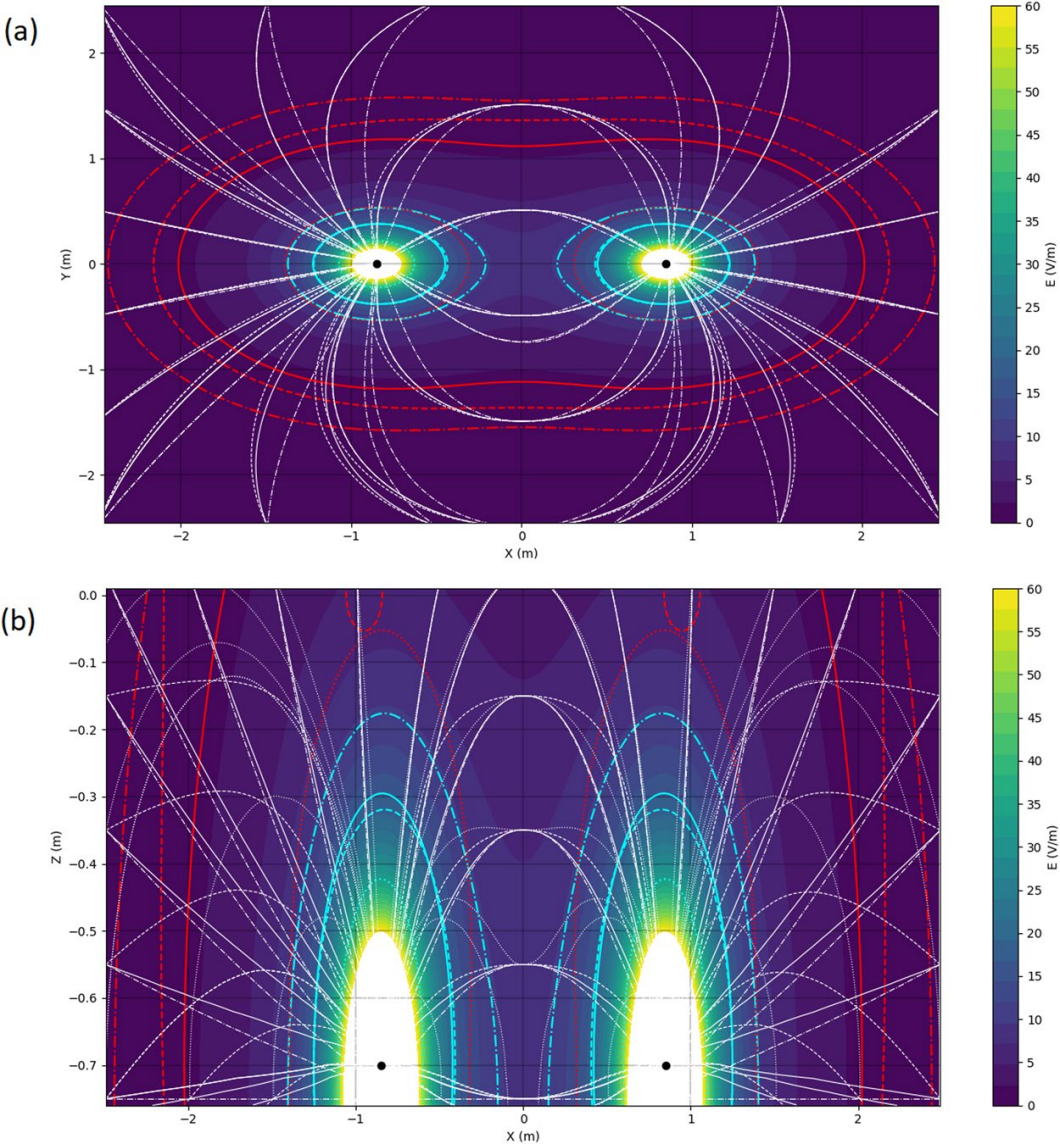
Theorical electric field strength overlayed for electrodes in Mid-water (full lines), close to the surface (dashed lines), close to a non-conductive seabed (dashed-dot lines), and close to a conductive seabed (dotted lines) in (**a**) horizontal plane (Z = -55 cm) and (**b**) vertical plane Y = 0 cm). Coloured lines represent the magnitude of the electric field (red: 1 Vm-1; cyan: 10 Vm-1); white lines represent the vector field lines for (**b**) and a projected vector field line on the horizontal plane for (**a**).

The models show that the electric field propagation was affected by the surface and seabed (Fig. 4). In both planes, the electric fields propagated slightly further when the Scuba7 was close to the surface and the seabed compared to a mid-water situation. For example, 1 Vm^−1^ was modelled ~1.1 m horizontally from the electrodes in mid-water, but ~1.5 m when close to the non-conductive seabed. However, this difference was not as pronounced close to the electrodes, with the field strength of the Scuba7 in mid-water and close to the surface being similar at ~40 cm and that of the Scuba7 close to the non-conductive seabed propagating only ~15 cm further. The propagation of the electric field was most affected by the conductivity of the seabed, with field strength at 50 cm being ~1 Vm^−1^ *vs*. ~10 Vm^−1^ for the model with non-conductive seabed.

**Figure 5:**
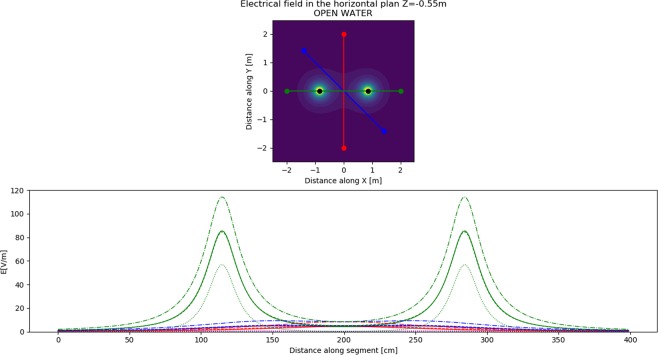
Field strength that sharks were exposed to when crossing through the dipole axis along (**a**) three different approach directions (parallel = green; perpendicular = red; diagonal = blue) and for (**b**) four model situations (Mid-water = full line; Surface = dashed line; non-conductive seabed = dashed-dot line; conductive seabed = dotted line). Note that dashed lines are difficult to see because they overlay on top of the full lines.

The field strength that sharks were exposed to varied substantially by the approach direction (Fig. 5). Sharks were exposed to a field <8 Vm^−1^ when approaching perpendicularly or diagonally to the dipole axis and passing in-between the electrodes. However, electric field exposure increased to 80 Vm^−1^ as sharks swam on top of the electrodes. The increased electric field exposure was more pronounced when the Scuba7 was close to the seabed and non-conductive, nearly reaching an exposure of 115 Vm^−1^. The field strength only reached 60 Vm^−1^ when the seabed was conductive.

## Discussion

The global rise in shark attacks has prompted the development of new methods to prevent or reduce the risk and injuries from shark bites. While several studies have previously tested the effects of electric shark deterrents^12,15,16,39^, this is the first focused on a small species, the blacktip reef sharks, with many sharks present simultaneously, and undertaken in shallow water (<1 m). Blacktip reef sharks exhibited an obvious behavioural change when exposed to an active Scuba7, with sharks remaining further away from the bait, which was not consumed as often. This resulted in an increased number of attempts to consume the bait. However, blacktip reef sharks were still able to come within close proximity of the active deterrent (80.98 ± 1.72 cm) and consumed the bait in 33% of the trials. This only occurred when sharks approached the bait from in-between the electrodes. Theoretical modelling of the electric field supported these findings with sharks being exposed to weak electrical fields when approaching perpendicularly and in-between electrodes. Testing of the Kevlar-neoprene showed that in situations when personal deterrents do not prevent bites, injuries can be reduced through puncture-resistant fabric. The number and size of holes were all reduced by the Kevlar layer. Combined, these results show that the risk and injuries from blacktip reef sharks can be reduced by the use of personal deterrents and puncture-resistant fabric.

Whilst most studies have tested deterrents on individual sharks^12,15,16^, this study tested an electric deterrent around up to 30 sharks at any given time. Previous studies have shown that competition can change behaviour and hierarchy in sharks^20^ and that high shark densities can result in non-selective mob rushes towards the bait and diminish the effectiveness of shark deterrents^21^. However, the number of sharks present in our study only affected the number of approaches, i.e. more sharks led to more approaches, but did not otherwise hamper the efficacy of the Scuba7. This either suggests that competition does not change the effects of electric deterrents on blacktip reef sharks or that competition did not occur in the situation tested. As testing was conducted in an area where sharks are frequently fed by locals, the regular food supply may have also reduced the need for competition.

Our results confirm a previous study showing that although an electric pulse can deter sharks from consuming a bait or breaching on an intended prey, it cannot prevent all bites^12,15,16^. In the present study, blacktip reef sharks only consumed the bait after approaching perpendicular and in-between to the dipole axis. This only occurred when the water current was running perpendicular to the axis of the electrodes and sharks followed the scent in that direction. This direction of approach allowed sharks to come closer to the bait without swimming directly over the electrodes. When sharks approached from a different direction, they were more likely to swim over the electrodes to reach the bait and be deterred when in close proximity to the electrodes. The theoretical models support this, with sharks only being exposed to a strong electrical field (>80 Vm^−1^) when swimming over the electrodes, while not being exposed to fields >8 Vm^−1^ when approaching perpendicularly or diagonally and in-between the electrodes. This is likely due to electric field strength rapidly decreasing with distance^40^, with the strength of electric field diminishing from ~115 Vm^−1^ at the electrodes to 7–16 Vm^−1^ when 80 cm away from the electrodes^16,41^. These values were obtained by measuring electric fields produced by various Ocean Guardian products (i.e. Freedom7, Surf+ Freedom7) placed at the surface in a 4 m deep sheltered bay^16^ and in a 1.2 m deep tank^41^, and are similar to values obtained from our theoretical models. While our models show that proximity to the surface and seabed can affect electric field propagation, these effects are relatively small close to the electrodes where sharks are most deterred. This highlights the importance of adequate electrode placement as the ability to deter sharks will depend on the strength of the electric field which is most strongly affected by the distance to the electrodes^12^. Overall, this study shows that electric fields have a similar ability to reduce bait consumption in blacktip reef sharks and white sharks. The similar behavioural response across the two species was observed regardless of the water depth difference between the two studies and proximity of the seabed in the present study. This might be expected as many elasmobranch species have a similar electrosensory threshold sensitivity^42^. However, detection threshold and behavioural response to electric fields can vary extensively between species^43–45^. For example, maximum voltage gradient tolerated by *Sphyrna lewini* is 18.5 Vm^−1^ and is higher than the deterrent threshold suggested for *Triakis semifasciata* (9.6 Vm^−1^), *C. obscurus* (7–10 Vm^−1^), and *C. leucas* (3 Vm^−1^)^44,46^. Results from this study should, therefore, not be extrapolated to other species or situations and further testing of electric field-based shark deterrents on other potentially dangerous species (e.g., tiger shark, *Galeocerdo cuvier*; bull shark *C. leucas*) is recommended.

The Kevlar layer glued to the neoprene reduced damages to the neoprene and therefore, Kevlar-neoprene has the potential to minimise injuries and blood loss from shark bites. While the reduced damaged is likely due to the Kevlar layer, it is possible that the method of attachment, i.e. glued on the neoprene, contributed to the reduction of punctures by decreasing neoprene flexibility. Our results also found no differences in the number of punctures and puncture size between thick (5 mm) and thin (3 mm) neoprene. This suggests that a thin layer of Kevlar could be used for protection from shark bites without unnecessarily using a thick layer of neoprene, thus promoting useability and wearability of the Kevlar-neoprene material in a range of applications. Most aquatic activities using wetsuits (e.g., surfing, snorkelling, diving) requires the material to be light and flexible. Although the Kevlar-neoprene is lighter and more practical than the chainmail suit, its suitability across various activities remains to be tested, but the use of a puncture-resistant layer glued or incorporated to standard neoprene material might bridge the gap between currently used wetsuits and the heavy and relatively impractical chainmail suit.

We are cognisant that the Kevlar-neoprene will not prevent or lessen bone breakages and crushing injuries. However, the primary cause of death in fatal shark bites is blood loss caused from vascular lacerations^47–50^. The Kevlar-neoprene could therefore still prove effective in reducing haemorrhaging by providing additional time for a shark bite victim to be attended to by emergency services, thus reducing the likelihood of death^48,49^. Furthermore, many shark bites around the world are inflicted by small shark species (e.g., blacktip sharks *C. limbatus*; spinner sharks *C. brevipinna*; Caribbean reef sharks *C. perezii;* silky sharks *C. falciformis*)^51^ that are less likely to cause crushing injuries but are capable of inflicting damages to tissues, ligaments, and nerves^52^. The ability of Kevlar-neoprene to reduce injuries will differ between species and will be affected by species size, jaw strength, teeth morphology, and bite kinematics. Similarly to the need to test electric field-based deterrents on a range of species, testing Kevlar-neoprene on other potentially dangerous species (e.g., white shark, *Carcharodon carcharias*; tiger shark; bull shark) is warranted.

Whilst many studies focus on bites from large predatory sharks, this study is relevant to smaller sharks, which are also responsible for many shark bites worldwide^1^. For example, Florida has the highest number of incidents, with most bites likely to be from blacktip or spinner sharks^3^. Our findings are also relevant to wildlife tourism and shark feeders who often use gloves or to spearfishers who are also victims of bites from small sharks. We also acknowledge that we did not test damages to skin, muscle, or other material mimicking those, which would normally be under the neoprene. We instead infer that reduced punctures or tear in the Kevlar-neoprene compared to standard neoprene is likely to result in reduced injuries to a person wearing this material. Future studies should validate this assumption.

## Conclusion

The global increase in number of shark bites has led to the development of a range of shark-mitigation measures and personal deterrents rapidly becoming commercially available. Our study unambiguously shows that electric field-based deterrents can change the behaviour of sharks and reduce shark bite risk. In cases when a shark bite still occur, a Kevlar layer glued to neoprene reduces punctures and might therefore reduce injuries and blood loss. Our results will enable private and government agencies to make informed decisions about these devices and to implement shark bite mitigation strategies. Our findings will also inform ocean users and allow them to decide which product might be most suitable.

## Supplementary information

Supplementary information.

## Data Availability

The datasets generated and analysed during the current study are available from the corresponding author on reasonable request.
