# Supplementary material for: Response of blacktip reef sharks Carcharhinus melanopterus to shark bite mitigation products
Source: Sci Rep. 2020 Feb 27;10:3563. doi: 10.1038/s41598-020-60062-x (PMC7046715; doi:10.1038/s41598-020-60062-x)
Supplement: Supplementary file 1 — Supplementary information. [file 41598_2020_60062_MOESM1_ESM.docx]

Supplementary material

Response of blacktip reef sharks *Carcharhinus melanopterus* to shark bite mitigation products

Madeline Thiele^1^, Johann Mourier^2, 3, 4^, Yannis Papastamatiou^5^, Laurent Ballesta^6^, Eric Chateauminois^7^, Charlie Huveneers^1,*^

^1^ Southern Shark Ecology Group, College of Science and Engineering, Flinders University, Bedford Park, SA, 5042, Australia

^2^ MARBEC, Univ Montpellier, CNRS, IFREMER, IRD, Sète, France

^3^ EPHE, PSL Research University, CRIOBE USR3278 EPHECNRS-UPVD, 66860 Perpignan, France

^4^ Labex Corail, CRIOBE, 98729 Moorea, French Polynesia

^5^ Department of Biological Sciences, Florida International University, North Miami, Florida, USA

^6^ Andromede Oceanologie, Place Cassan, 34280 Carnon, France

^7^ Shark Security Centre, 2 Quai Gilbert, 97460 Saint Paul, Reunion Island, France

* Corresponding author: [Charlie.huveneers@flinders.edu.au](mailto:Charlie.huveneers@flinders.edu.au)

**Table S1. Distance model variants.** Generalised linear mixed-effects model (GLMM) results of models (from top-ranked [indicated in bold] to worst) estimating the effects of the Scuba7 on distance to the bait. An interaction between variables is indicated by ‘*’. The df = degrees of freedom, LL = log likelihood, AIC_c_ = Akaike’s Information Criterion corrected for small sample size, dAIC_c_ = difference in AIC_c_ between the current and the top-ranked model, wAIC_c_ = model probability.

| Model | (Int) | Pass orientation | Status | Pass orientation* Status | df | LL | AIC_c_ | dAIC_c_ | wAIC_c_ |
| --- | --- | --- | --- | --- | --- | --- | --- | --- | --- |
| 8 | **3.46** | **X** | **X** | **X** | **6** | **-2386.08** | **4784.2** | **0** | **0.80** |
| 4 | 3.86 | X | X |  | 5 | -2388.47 | 4787.0 | 2.76 | 0.20 |
| 16 | 1.30 | X | X | X X | 6 | -2408.77 | 4829.6 | 45.38 | 0 |
| 12 | 1.68 | X | X | X | 5 | -2410.93 | 4831.9 | 47.68 | 0 |
| 3 | 5.07 |  | X |  | 4 | -2435.31 | 4878.7 | 94.42 | 0 |
| 2 | 5.78 | X |  |  | 4 | -2444.14 | 4896.3 | 112.08 | 0 |
| 11 | 2.88 |  | X | X | 4 | -2454.39 | 4916.8 | 132.57 | 0 |
| 10 | 3.62 | X |  | X | 4 | -2464.89 | 4937.8 | 153.57 | 0 |
| 1 | 7.98 |  |  |  | 3 | -2513.64 | 5033.3 | 249.06 | 0 |
| 9 | 5.79 |  |  | X | 3 | -2530.20 | 5066.4 | 282.19 | 0 |

**Table S2. Time model variants.** Generalised linear mixed-effects model (GLMM) results of models (from top-ranked [indicated in bold] to worst) estimating the effects of the Scuba7 on distance to the bait. An interaction between variables is indicated by ‘*’. The off(log(MxN)) = maximum number of sharks as offset, df = degrees of freedom, LL = log likelihood, AIC_c_ = Akaike’s Information Criterion corrected for small sample size, dAIC_c_ = difference in AIC_c_ between the current and the top-ranked model, wAIC_c_ = model probability.

| Model | (Int) | Status | off(log(MxN)) | df | LL | AIC_c_ | dAIC_c_ | wAIC_c_ |
| --- | --- | --- | --- | --- | --- | --- | --- | --- |
| 2 | **-0.82** | **X** |  | **4** | **-74.204** | **156.9** | **0** | **1** |
| 4 | -2.90 | X | X | 4 | -88.752 | 186.0 | 29.1 | 0 |
| 1 | 0.00 |  |  | 3 | -119.987 | 246.3 | 89.36 | 0 |
| 3 | -2.05 |  | X | 3 | -129.984 | 266.3 | 109.35 | 0 |

**Table S3. Number of approaches model variants.** Generalised linear mixed-effects model (GLMM) results of models (from top-ranked [indicated in bold] to worst) estimating the effects of the Scuba7 on distance to the bait. An interaction between variables is indicated by ‘*’. The off(log(MxN)) = maximum number of sharks as offset, df = degrees of freedom, LL = log likelihood, AIC_c_ = Akaike’s Information Criterion corrected for small sample size, dAIC_c_ = difference in AIC_c_ between the current and the top-ranked model, wAIC_c_ = model probability.

| Model | (Int) | Status | off(log(MxN)) | df | LL | AIC_c_ | dAIC_c_ | wAIC_c_ |
| --- | --- | --- | --- | --- | --- | --- | --- | --- |
| 4 | **-1.178** | **X** | **X** | **4** | **-105.19** | **218.9** | **0** | **0.999** |
| 2 | 0.90 | X |  | 4 | -112.43 | 233.4 | 14.47 | 0.001 |
| 3 | -0.29 |  | X | 3 | -137.68 | 281.7 | 62.77 | 0 |
| 1 | 1.76 |  |  | 3 | -138.65 | 283.6 | 64.7 | 0 |

**Table S4. Bait consumption model variants.** Generalised linear mixed-effects model (GLMM) results of models (from top-ranked [indicated in bold] to worst) estimating the effects of the Scuba7 on distance to the bait. An interaction between variables is indicated by ‘*’. The off(log(MxN)) = maximum number of sharks as offset, df = degrees of freedom, LL = log likelihood, AIC_c_ = Akaike’s Information Criterion corrected for small sample size, dAIC_c_ = difference in AIC_c_ between the current and the top-ranked model, wAIC_c_ = model probability.

| Model | (Int) | Status | off(log(MxN)) | df | LL | AIC_c_ | dAIC_c_ | wAIC_c_ |
| --- | --- | --- | --- | --- | --- | --- | --- | --- |
| 2 | **22.34** | **X** |  | **3** | **-23.52** | **53.3** | **0** | **0.95** |
| 4 | 20.4 | X | X | 3 | -26.44 | 59.2 | 5.85 | 0.05 |
| 1 | 0.71 |  |  | 2 | -53.34 | 110.8 | 57.49 | 0 |
| 3 | -1.26 |  | X | 2 | -58.23 | 120.6 | 67.28 | 0 |

**Table S5. Reaction model variants.** Generalised linear mixed-effects model (GLMM) results of models (from top-ranked [indicated in bold] to worst) estimating the effects of the Scuba7 on distance to the bait. An interaction between variables is indicated by ‘*’. The off(log(MxN)) = maximum number of sharks as offset, df = degrees of freedom, LL = log likelihood, AIC_c_ = Akaike’s Information Criterion corrected for small sample size, dAIC_c_ = difference in AIC_c_ between the current and the top-ranked model, wAIC_c_ = model probability.

| Model | (Int) | Pass distance | Pass orientation | Status | Pass orientation*Status | off(log(MxN)) | df | LL | AIC_c_ | dAIC_c_ | wAIC_c_ |
| --- | --- | --- | --- | --- | --- | --- | --- | --- | --- | --- | --- |
| 16 | **-1.53** | **-0.074** | **X** | **X** | **X** |  | **6** | **-174.92** | **362.0** | **0** | **0.87** |
| 8 | -1.11 | -0.071 | X | X |  |  | 5 | -177.96 | 366.1 | 4.02 | 0.12 |
| 32 | -3.73 | -0.075 | X | X | X | X | 6 | -179.68 | 371.6 | 9.52 | 0.01 |
| 6 | -1.32 | -0.073 |  | X |  |  | 4 | -182.26 | 372.6 | 10.57 | 0 |
| 24 | -3.32 | -0.072 | X | X |  | X | 5 | -182.39 | 374.9 | 12.88 | 0 |
| 22 | -3.59 | -0.075 |  | X |  | X | 4 | -188.41 | 384.9 | 22.87 | 0 |
| 15 | -2.14 |  | X | X | X |  | 5 | -198.23 | 406.6 | 44.57 | 0 |
| 7 | -1.80 |  | X | X |  |  | 4 | -199.99 | 408.1 | 46.04 | 0 |
| 31 | -4.35 |  | X | X | X | X | 5 | -202.57 | 415.3 | 53.25 | 0 |
| 23 | -4.02 |  | X | X |  | X | 4 | -204.38 | 416.9 | 54.81 | 0 |
| 5 | -2.14 |  |  | X |  |  | 3 | -206.75 | 419.6 | 57.51 | 0 |
| 21 | -4.44 |  |  | X |  | X | 3 | -213.56 | 433.2 | 71.14 | 0 |
| 2 | 0.437 | -0.01 |  |  |  |  | 3 | -272.43 | 550.9 | 188.86 | 0 |
| 1 | 0.149 |  |  |  |  |  | 2 | -273.85 | 551.7 | 189.68 | 0 |
| 4 | 0.450 | -0.01 | X |  |  |  | 4 | -272.41 | 552.9 | 190.88 | 0 |
| 3 | 0.218 |  | X |  |  |  | 3 | -273.70 | 553.5 | 191.41 | 0 |
| 18 | -1.78 | -0.01 |  |  |  | X | 3 | -275.89 | 557.8 | 195.79 | 0 |
| 17 | -2.09 |  |  |  |  | X | 2 | -277.55 | 559.1 | 197.08 | 0 |
| 20 | -1.72 | -0.01 | X |  |  | X | 4 | -275.68 | 559.5 | 197.41 | 0 |
| 19 | -1.96 |  | X |  |  | X | 3 | -276.98 | 560.0 | 197.98 | 0 |

**Table S6. Size of holes model variants.** Generalised linear mixed-effects model (GLMM) results of models (from top-ranked [indicated in bold] to worst) estimating the effects of the Scuba7 on distance to the bait. An interaction between variables is indicated by ‘*’. The df = degrees of freedom, LL = log likelihood, AIC_c_ = Akaike’s Information Criterion corrected for small sample size, dAIC_c_ = difference in AIC_c_ between the current and the top-ranked model, wAIC_c_ = model probability.

| Model | (Int) | Thickness | Type | Thickness*Type | df | LL | AIC_c_ | dAIC_c_ | wAIC_c_ |
| --- | --- | --- | --- | --- | --- | --- | --- | --- | --- |
| 8 | **3.77** | **X** | **X** | **X** | **6** | **-3131.24** | **6274.6** | **0** | **0.66** |
| 4 | 4.21 | X | X |  | 5 | -3133.00 | 6276.1 | 1.51 | 0.31 |
| 3 | 3.77 |  | X |  | 4 | -3136.44 | 6280.9 | 6.37 | 0.03 |
| 2 | 6.05 | X |  |  | 4 | -3140.74 | 6289.5 | 14.95 | 0 |
| 1 | 5.60 |  |  |  | 3 | -3144.13 | 6294.3 | 19.71 | 0 |

**Table S7. Number of holes model variants.** Generalised linear mixed-effects model (GLMM) results of models (from top-ranked [indicated in bold] to worst) estimating the effects of the Scuba7 on distance to the bait. An interaction between variables is indicated by ‘*’. The df = degrees of freedom, LL = log likelihood, AIC_c_ = Akaike’s Information Criterion corrected for small sample size, dAIC_c_ = difference in AIC_c_ between the current and the top-ranked model, wAIC_c_ = model probability.

| Model | (Int) | Thickness | Type | Thickness*Type | df | LL | AIC_c_ | dAIC_c_ | wAIC_c_ |
| --- | --- | --- | --- | --- | --- | --- | --- | --- | --- |
| 3 | **14.92** |  | **X** |  | **3** | **-103.175** | **213.7** | **0** | **0.76** |
| 4 | 12.87 | X | X |  | 4 | -103.11 | 216.6 | 2.89 | 0.18 |
| 8 | 18.17 | X | X | X | 5 | -102.546 | 218.8 | 5.16 | 0.06 |
| 1 | 41.82 |  |  |  | 2 | -112.107 | 228.8 | 15.16 | 0 |
| 2 | 42.67 | X |  |  | 3 | -112.101 | 231.5 | 17.85 | 0 |

**Table S8. Proportion of holes model variants.** Generalised linear mixed-effects model (GLMM) results of models (from top-ranked [indicated in bold] to worst) estimating the effects of the Scuba7 on distance to the bait. An interaction between variables is indicated by ‘*’. The df = degrees of freedom, LL = log likelihood, AIC_c_ = Akaike’s Information Criterion corrected for small sample size, dAIC_c_ = difference in AIC_c_ between the current and the top-ranked model, wAIC_c_ = model probability.

| Model | (Int) | Thickness | Type | Thickness*Type | df | LL | AIC_c_ | dAIC_c_ | wAIC_c_ |
| --- | --- | --- | --- | --- | --- | --- | --- | --- | --- |
| 8 | **0.01** | **X** | **X** | **X** | **5** | **37.26** | **-60.8** | **0** | **0.99** |
| 4 | -0.03 | X | X |  | 4 | 30.79 | -51.2 | 9.54 | 0.01 |
| 3 | 0.01 |  | X |  | 3 | 26.62 | -45.9 | 14.86 | 0 |
| 2 | 0.02 | X |  |  | 3 | 25.60 | -43.9 | 16.89 | 0 |
| 1 | 0.05 |  |  |  | 2 | 23.34 | -42.1 | 18.71 | 0 |
